# Optimized disease prediction in healthcare systems using HDBN and CAEN framework

**DOI:** 10.1016/j.mex.2025.103338

**Published:** 2025-04-25

**Authors:** G. Prabaharan, S.M. Udhaya Sankar, V. Anusuya, K. Jaya Deepthi, Rayappan Lotus, R. Sugumar

**Affiliations:** a Department of Computer Science and Engineering, Vel Tech Rangarajan Dr. Sagunthala R&D Institute of Science and Technology, Chennai, India; b Department of CSE (Cyber Security), R.M.K. College of Engineering and Technology, Puduvoyal, India; c Department of Information Technology, Ramco Institute of Technology, Rajapalayam, India; d Department of Artificial Intelligence and Machine Learning, School of Computing, Mohan Babu University, Tirupati, Andhra Pradesh, India; e Institute of CSE, SIMATS Engineering, Saveetha Institute of Medical and Technical Sciences (SIMATS), Thandalam, Chennai, India

**Keywords:** Adaptive ensemble learning, Disease prediction, Machine learning, Feature extraction, Dense neural networks, Classification, Segmentation, Hybrid Deep Belief Network (HDBN), dynamic prediction aggregation through a Custom Adaptive Ensemble Network (CAEN)

## Abstract

Classification and segmentation play a pivotal role in transforming decision-making processes in healthcare, IoT, and edge computing. However, existing methodologies often struggle with accuracy, precision, and specificity when applied to large, heterogeneous datasets, particularly in minimizing false positives and negatives. To address these challenges, we propose a robust hybrid framework comprising three key phases: feature extraction using a Hybrid Deep Belief Network (HDBN), dynamic prediction aggregation via a Custom Adaptive Ensemble Network (CAEN), and an optimization mechanism ensuring adaptability and robustness. Extensive evaluations on four diverse datasets demonstrate the framework’s superior performance, achieving 93 % accuracy, 87 % precision, 95 % specificity, and 91 % recall. Advanced metrics, including a Matthews Correlation Coefficient of 0.8932, validate its reliability. The proposed framework establishes a new benchmark for scalable, high-performance classification and segmentation, offering robust solutions for real-world applications and paving the way for future integration with explainable AI and real-time systems.

• Designed a novel hybrid framework integrating HDBN and CAEN for adaptive feature extraction and prediction.
• Proposed dynamic prediction aggregation and optimization strategies enhancing robustness across diverse data scenarios.

## Graphical abstract

**Figure:**
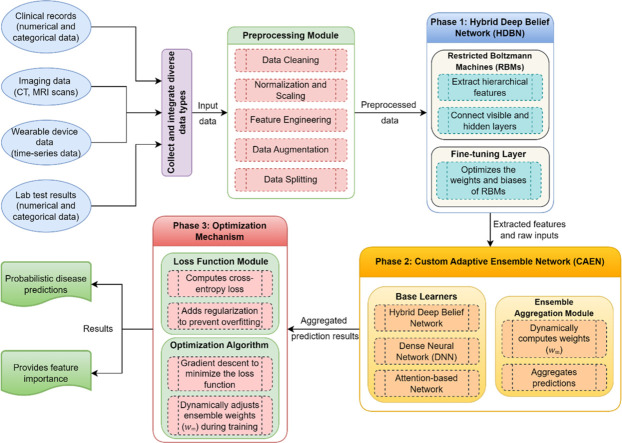

Specifications table

**Table:** 

Subject area:	Engineering
More specific subject area:	Improving decision-making regarding medical data analysis
Name of your method:	Hybrid Deep Belief Network (HDBN), dynamic prediction aggregation through a Custom Adaptive Ensemble Network (CAEN)
Name and reference of original method:	Adaptive Ensemble Network, Recursive Feature Elimination, and Dense Neural Networks
Resource availability:	*N.A.*

## Background

### Introduction

In the modern complexity of healthcare systems, the volume of healthcare data has grown tremendously: there are clinical records, imaging such as CT and MRI scans, wearable readings, and laboratory test results [1]. These data sources are very heterogeneous because they comprise numerical values, categorical attributes, time series information, and high-dimensional imaging. The data diversity this provides represents unparalleled possibilities to implement disease prediction or even diagnosis, thus being able to detect possible health issues in time, or even, as we are approaching it, to ensure personalized medicine [2]. Nevertheless, enormous challenges arise from making effective use of such data, due to underlying problems such as missing data, imbalanced datasets, noise, the amalgamation of various types of data modalities, etc. It is unclear whether disease prediction systems can be introduced in real-world clinical applications with sufficient flexibility to cope with the inherent complexity of these scenarios while maintaining fairness, interpretability, scalability, and robustness [[3], [4], [5]]. Many existing methods for disease prediction do not address well the nature of healthcare data which is multi-dimensional. However, traditional machine learning algorithms are powerful but have been found to struggle with dynamic aggregation of diverse data sources and are unable to generalize well against varied patient populations [[6], [7], [8]]. Furthermore, it was not adequate to extract features conventionally and to use methods from the ensemble class in general to accommodate hierarchical and temporal relationships contained in clinical as well as time series data. That implies designing advanced systems to bridge the difference between raw data handling and actionable insights. To address this critical gap, we propose a novel hybrid framework consisting of advanced deep-learning techniques and dynamic ensemble strategies to produce robust and accurate robust disease prediction [9].

The motivations of this research are in response to the increasing need for handling ever-increasing complexity in healthcare data, with reliable and scalable systems necessary. Usually, current predictive models restrict generalizability due to the limited of the generality when deployed on various datasets with different data types and with imbalance class distribution. Second, the requisite of personalized healthcare solutions makes it pertinent that models are to be adapted to new data and incorporate domain-specific knowledge. We wish to solve the problems by designing a system that will tackle these challenges, allowing us to improve prediction accuracy through the effective integration of different learning paradigms. However, instead of maintaining the current state of disease prediction systems, we seek to build a framework that integrates hierarchical feature extraction, dynamic ensemble learning, and robust optimization mechanisms to improve the state of disease prediction systems.

To overcome the challenges that we identified, in this work, we propose a three-phase framework that handles all aspects of preprocessing, modeling, and optimization in disease prediction comprehensively. So, the preprocessing phase is used during which rigorous cleaning, normalization, feature engineering, and augmentation take place to ensure data quality and consistency. Hierarchical features suggested by the underlying patterns and structures of the input data are extracted in the first phase through a Hybrid Deep Belief Network (HDBN). The second phase introduces a Custom Adaptive Ensemble Network (CAEN), a model that automatically aggregates diverse base learner predictions like Dense Neural Networks (DNNs) and attention-based networks using dynamic aggregation. In this phase, this ensemble’s predictive power is leveraged with a dynamic weighting mechanism which is optimized to maximize performance metrics. The optimization phase involves the use of a loss function designed with care and with regularization terms that prevent overfitting and increase generalizability. Through a systematic treatment of the whole predictive pipeline, the proposed framework addresses together the many challenges in disease prediction in complex healthcare settings.

### Related work

Much progress has been made in the field of classification and segmentation models, but many remained unsolved. Model accuracy vs computational efficiency trade-off is one of the major concerns, when you have large scale data [10]. However, traditional deep learning models typically face difficulties with high dimensional data and are thus more time consuming as well as their feature extraction is under optimal. In fact, it remains a challenging problem to guarantee model generalization on a variety of datasets as conventional approaches have a tendency to overfit particular data distributions thus have bad flexibility to new situations. Recent works have explored different strategies to address these limitations. Hybrid deep learning models have gained traction by integrating multiple architectures to enhance feature learning and classification accuracy [[11], [12], [13]]. For instance, methods like SKR-DMKCF and DJ-RNN have introduced customized network structures to improve prediction consistency. However, these models still suffer from high false positive and false negative rates, limiting their reliability. Additionally, ensemble learning techniques, such as those employed in Hsc-AN and CSD, have been proposed to combine multiple classifiers for improved decision-making. While these approaches offer enhanced robustness, their reliance on predefined aggregation strategies often results in performance bottlenecks when dealing with highly dynamic datasets.

Recent advancements are those based on adaptive and optimization driven methodologies, which are effective to overcome these challenges. A promising method of optimizing resource allocation and dynamic feature selection which has been explored so far is based on techniques that involve reinforcement learning, continual learning, and evolutionary algorithms. Nevertheless, such approaches are usually accompanied by extra computational overhead resulting in their inapplicability for real-time applications. Anish et al. [14] introduced a hybrid cascaded deep learning framework that integrates ensemble feature selection and feature fusion mechanisms for multi-disease prediction. The feature selection is optimized using the MSB-EV), which selects the most informative features from statistical, deep, and optimally weighted features. The final classification step employs an HSC-AttentionNet, combining a Deep Temporal Convolution Network and LSTM for sequential data processing. The proposed optimization further enhances model robustness, leading to superior predictive performance.

Naveen Reddy et al. [15] focused on healthcare data preprocessing and feature extraction using a Weighted Convolutional Neural Network Feature with Dilated Gated Recurrent Unit (WCNNF-DGRU). The preprocessing pipeline includes data cleaning, transformation, and outlier detection via Density-Based Spatial Clustering of Applications with Noise. The extracted features are refined using the Enhanced Kookaburra Optimization Algorithm, ensuring optimal weight allocation within the CNN structure for improved disease prediction accuracy. Kalpana et al. [16] addressed image preprocessing challenges with Advanced Image Preprocessing Techniques (AIPT), integrating Adaptive Thresholding, Dynamic Histogram Equalization, Hierarchical Contrast Normalization, Multi-Scale Region Enhancement, and Contextual Feature Augmentation. This preprocessing framework enhances image quality by reducing noise and preserving structural details. Their subsequent work [17] introduced the Synergistic Kruskal-RFE Selector as well as the distributed multi-kernel classification framework. The model combines Recursive Feature Elimination (RFE) and multi-kernel classification, enabling efficient dimensionality reduction while maintaining essential discriminatory features for classification.

Sathya et al. [18] proposed an ensemble classification framework leveraging AdaBoost, Support Vector Classifier (SVC), and decision trees to enhance the accuracy of universal disease prediction. Their approach utilizes a voting mechanism to combine individual classifiers, ensuring a robust decision-making process. By incorporating diverse classification paradigms, the framework adapts to different disease types, thereby improving prediction reliability. Anish et al. [19] introduced a Deep Jordan Recurrent Neural Network for multi-coronary heart disease (Multi-CHD) prediction using 3D CT scan images. The preprocessing phase applies Scaled Functionality-centric Contrast Limited Adaptive Histogram Equalization (SF-CLAHE) to enhance image contrast. The segmented images are processed using the RegulaFalsi Hard C-Means (RFHCM) algorithm for blood pool and heart chamber segmentation. Additionally, a Supervised Descent Method (SDM) extracts vessel structures, and feature selection is performed using Weight Factor-based Tunicate Swarm Optimization (WF-TSO). The extracted features are then classified using the proposed deep learning framework, demonstrating improved segmentation and classification performance. Prabaharan et al. [20] developed an AI-driven liver tumor prediction framework, integrating multiple feature domains such as histology, genomics, radiomics, videomics, and clinical data. The framework employs a Histological Convolutional Autoencoder (HistoCovAE) for detailed tumor segmentation and a Genomic Feature Extraction module (MIRSLiC) for molecular marker analysis. This comprehensive feature integration allows for precise tumor subtype classification and prognosis prediction.

Gupta et al. [21] leveraged a genetic algorithm-based recursive feature elimination (GA-RFE) technique combined with AdaBoost for predicting lifestyle diseases such as diabetes and cardiovascular conditions. Their approach refines feature selection iteratively, enhancing model interpretability while reducing computational overhead. The framework was tested using Cleveland and Pima datasets, demonstrating improved classification accuracy. Kshitija et al. [22] explored a multi-faceted ML framework integrating SVM, decision trees, and deep neural networks to analyze heterogeneous medical data. Their model emphasizes feature selection and dimensionality reduction to improve interpretability while maintaining high predictive accuracy. The study highlights the significance of integrating multiple data sources, including medical records, genomic data, and lifestyle indicators, to enhance disease prediction capabilities. Mathews et al. [23] introduced a web-based multi-disease prediction system designed for real-time risk assessment. Their approach integrates statistical models with deep learning techniques to process user-provided health data, including symptoms and medical history. The system employs disease-specific models trained on diverse medical datasets, enabling accurate and accessible disease risk evaluation. Fakhrud et al. [24] proposed a flexible and scalable medical diagnostic framework utilizing both ML and DL techniques to predict multiple diseases simultaneously. The framework aims to address issues of misdiagnosis and delayed detection by leveraging an integrated data analysis pipeline. By streamlining multi-disease prediction into a single platform, the study demonstrates the potential to reduce healthcare costs and improve accessibility.

Visumathi et al. [25] developed a Naive Bayesian network-based multi-disease prediction model. The system applies statistical preprocessing and probabilistic classification to provide reliable disease predictions across various medical conditions. Their work emphasizes the affordability and computational efficiency of Bayesian approaches, making them suitable for large-scale deployment in healthcare applications. Arsath et al. [26] introduced a deep learning-based multi-disease prediction framework that integrates CNN and RNN architectures to capture both spatial and temporal dependencies in medical data. Their model focuses on kidney disease, heart disease, diabetes, and malaria prediction, demonstrating the adaptability of DL models to multiple medical domains. Kalaivani et al. [27] conducted a comprehensive review of existing ML and DL techniques for multi-disease classification. Their work outlines statistical methodologies, dataset characteristics, feature extraction strategies, and evaluation metrics used in prior studies. In addition, future research directions are proposed based on explainability, real-time deployment, and integration of multi-modal data. Karwa et al. [28] propose a disease diagnosis system based on Random Forest (RF), Naive Bayes, and SVM classifiers. The system they developed is meant to analyze patient symptoms as well as make probabilistic disease predictions. The study highlights the importance of ML-driven diagnostic tools in modern healthcare and in particular, in early diagnosis and intervention. Yazdi et al. [29] employed a novel integration of metaheuristic algorithms, ANFIS, and K-means clustering to evaluate the performance of countries during the COVID-19 pandemic. This study highlighted the importance of combining diverse techniques to uncover intricate patterns and improve decision-making, setting a foundation for adopting advanced hybrid models in healthcare. Exploring the potential of Explainable Artificial Intelligence (XAI) in clinical settings, Rane et al. [30] reviewed the role of transparent AI models across various medical domains, including radiology, pathology, cardiology, and oncology. The study underscored the critical need for interpretability in medical AI systems, with specific focus on enhancing clinician trust through techniques such as attention mechanisms and saliency maps. By demystifying AI decisions, this approach facilitates seamless integration into clinical workflows, paving the way for broader adoption of AI in healthcare.

Otoum et al. [31] investigated federated learning frameworks for healthcare data, utilizing TabNet’s architecture and blockchain technology to improve data handling and governance. The study integrated horizontal federated learning for secure model aggregation and incorporated smart contracts to automate transparency and accountability. These advancements demonstrate the potential of federated systems to facilitate secure multi-party collaborations while addressing the challenges of data privacy and interoperability in healthcare. Swami et al. [32] delved into the application of machine learning for analyzing datasets of multiple diseases, such as dengue and COVID-19, to support medical decision-making. By exploring the impact of various diseases through large-scale data analysis, the study emphasized the transformative role of AI in predictive analytics, aiding the medical industry in understanding complex patterns for early intervention and management. Singh et al. [33] proposed a lightweight convolutional neural network (CNN)-based healthcare model capable of real-time disease prediction in IoT-enabled environments. The integration of the EO-LWAMCNet model into a cloud-based IoT ecosystem illustrated its capability to revolutionize remote healthcare monitoring. By enhancing early diagnosis and expanding access to quality care, this approach underscores the potential of lightweight AI models in resource-constrained settings.

Despite progress in multi-disease prediction, there remain several challenges that are not addressed. Most of the existing works have tackled feature selection, dimensionality reduction, or classifier performance optimization separately. Yet, there are still few holistic frameworks such that advanced feature extraction can seamlessly be executed on heterogeneous medical datasets, based on robust preprocessing as well as combining modalities. However, most of the studies are limited to one type of disease or one data modality, which limits their genericity concerning more general healthcare applications. Additionally, there are other issues concerning model interpretability, practicability in real-time adaptability, and scalability for large-scale deployment that have not been exploited to a good extent. Applications of current optimization techniques to large and/or highly imbalanced datasets typically digress towards a tradeoff between computational efficiency and prediction accuracy. In addition, the establishment of explainable AI (XAI) methods to improve transparency in making diagnostic decisions is unaddressed as well, which results in the absence of full usability and trust in the clinical use of these systems. To fill these gaps disease prediction models, need to be developed that leverage cutting-edge deep networks, optimization strategies, and real-time processing capabilities and are unified, adaptable, and interpretable.

To address the identified research gaps, our proposed work introduces an innovative and unified framework that seamlessly integrates advanced techniques for multi-disease prediction. The approach begins with a robust preprocessing pipeline that handles heterogeneous medical datasets using adaptive methods such as hierarchical contrast normalization, contextual feature augmentation, and multi-scale region enhancement. This ensures data quality and consistency, reducing noise and preserving critical details across diverse modalities. For feature extraction, we employ a Hybrid Deep Belief Network (HDBN) that combines the strengths of generative models and deep learning to extract hierarchical and representative features from complex datasets. This is complemented by a Custom Adaptive Ensemble Network (CAEN) designed for dynamic prediction aggregation, ensuring adaptability and robustness across various disease types. The ensemble employs a voting mechanism to integrate multiple classifiers, balancing predictive accuracy and computational efficiency. To optimize feature selection and model parameters, we integrate evolutionary optimization techniques tailored for high-dimensional datasets. This ensures computational efficiency while maintaining superior predictive performance. Additionally, explainable AI (XAI) methods are embedded within the framework to provide transparent and interpretable decision-making insights, addressing the usability challenges in clinical applications. Our framework is scalable, adaptable, and capable of real-time deployment, overcoming limitations in generalizability, efficiency, and interpretability observed in existing works. This comprehensive solution positions our model as a pioneering step toward addressing the multifaceted challenges of multi-disease prediction.

### Method details

The proposed work entails designing a multi-phase approach for improving disease prediction accuracy by combining both sophisticated deep learning and adaptive ensemble approaches. The system takes in different data sources, which come from clinical records, imaging data (e.g., CT, MRI), wearable device data, and laboratory results, to be preprocessed to deal with missing values, scale normalization, and feature extraction. First, this architecture has 2 major phases: feature extraction using a Hybrid Deep Belief Network (HDBN); dynamic prediction aggregation using a Custom Adaptive Ensemble Network (CAEN); and an optimization phase to achieve model robustness and adaptation. Data processing through each phase is well interconnected and delivers highly efficient and accurate predictions for the same. The first step in the process is data preprocessing that is the input data is clean and balanced and the data is from the problem domain. In Phase 1, the HDBN is used for unsupervised and supervised feature learning to understand hierarchical patterns in the input data. These features are passed to the CAEN in Phase 2, which dynamically aggregates predictions from multiple base learners, including HDBN, Dense Neural Networks (DNN), and attention-based networks. Finally, Phase 3 optimizes the entire ensemble using cross-entropy loss with regularization to prevent overfitting, leveraging adaptive weight adjustments to refine the contributions of each component. This comprehensive framework is designed to provide robust and interpretable predictions, making it a significant contribution to the field of disease prediction.

### Process flow of the proposed work

The proposed framework for disease prediction follows a structured and systematic process flow, beginning with input data acquisition and progressing through preprocessing, feature extraction, dynamic aggregation, and optimization. Initially, diverse sources such as clinical records, imaging data, wearable device readings, and laboratory results are gathered. These data inputs, characterized by mixed types including numerical, categorical, time-series, and images, are preprocessed to address missing values, noise, and imbalanced classes. The preprocessing steps involve cleaning, normalization, feature engineering, and augmentation, ensuring the data is ready for analysis. This comprehensive preprocessing guarantees consistency and reliability, forming a robust foundation for further modeling. The Hybrid Deep Belief Network (HDBN) is utilized for feature extraction. Through an unsupervised pretraining process leveraging Restricted Boltzmann Machines (RBMs) and subsequently supervised fine-tuning, the HDBN captures hierarchical features that effectively represent the input data. These extracted features serve as the foundation for the Custom Adaptive Ensemble Network (CAEN).

The Custom Adaptive Ensemble Network (CAEN) integrates multiple base learners. Predictions from the HDBN, Dense Neural Network (DNN), and attention-based networks are aggregated dynamically using a weighted ensemble strategy. At this stage, a decision is made based on the performance metrics of the base learners to dynamically adjust the weights for aggregation. This ensures that the most relevant features and patterns are highlighted, leading to precise and accurate predictions. If the weights do not meet the desired performance threshold, they are recalibrated iteratively to enhance ensemble effectiveness. The optimization mechanism fine-tunes the system to ensure robustness and minimize errors. The process employs the cross-entropy loss function, combined with regularization terms, to prevent overfitting and enhance generalizability. A decision is made based on the evaluation of loss function values to adjust the ensemble weights dynamically. If the loss is above a defined threshold, the adaptive gradient descent algorithm continues to refine the weights. Once the loss meets the acceptable level, the optimization process is concluded. This process flows in a structured and iterative manner which guarantees that the proposed system proposes intricate relationships in the data, adapts to its complexity, and provides very accurate predictions. The use of decision making which is integrated at some critical stages can contribute to making the system more adaptable and accurate at the same time, thus the solution to the disease prediction is comprehensive and innovative. A summary of all symbols and their meanings utilized in the proposed work is listed in Table 1.

**Table 1:** Notation and meanings.

Notation	Explanation
F	Dataset with Features
H	Hyperparameters
$f_{i}$	Feature
X	Data Point
μ	Mean
σ	Standard Deviation
Z	Transformed Feature Matrix
$O_{i}$	Observed Frequencies
$E_{i}$	Expected Frequencies
$v_{i}$ and $h_{j}$	Visible and Hidden Units
Z	Partition Function
η	Learning Rate
${\hat{y}}_{i}$	Predicted Output
$\lambda ∥W{∥}^{2}$	Regularization term to prevent Overfitting
${\hat{Y}}_{m}$	Prediction from the m^th^ Base Learner
$w_{m}$	Weight Assigned to the m^th^ Base Learner.
$L_{m}$	Loss of the m^th^ Base Learner
$y_{i}$	Ground Truth for Sample i
$\hat{Y}$	Final Prediction
$R(w_{m})$	Regularization Function
$Y_{pred}$	Predicted Outputs
$W_{l}$	Weights
$F_{HDBN}$	Extracted Hierarchical Features

### Input data

Integration of diverse and multi-faceted data sources is the cornerstone of our predictive disease modeling. Clinical records include the patient’s medical histories, diagnoses, prescriptions, other structure details, and so on. Imaging data, also in the form of computed tomography (CT) scans or magnetic resonance imaging (MRI), provides high-resolution information regarding anatomical and pathological changes and complements these. Moreover, continuous monitoring data from wearable devices, including step counts, heart rate, and sleep patterns, can also be added. Very importantly, laboratory results can add data such as blood glucose levels, lipid profiles, and other diagnostic markers. The input data has mixed data types. This includes numerical data — physiological like data or lab test values; categorical data — gender, symptoms, disease, name, etc.; temporal data — time series data; and image data which is high dimensional, high feature-rich data. Harmonizing these heterogeneous modalities into a unified dataset, and bringing them to a consistency so that downstream processing can be carried out on them.

### Preprocessing

**Fig. 1:**
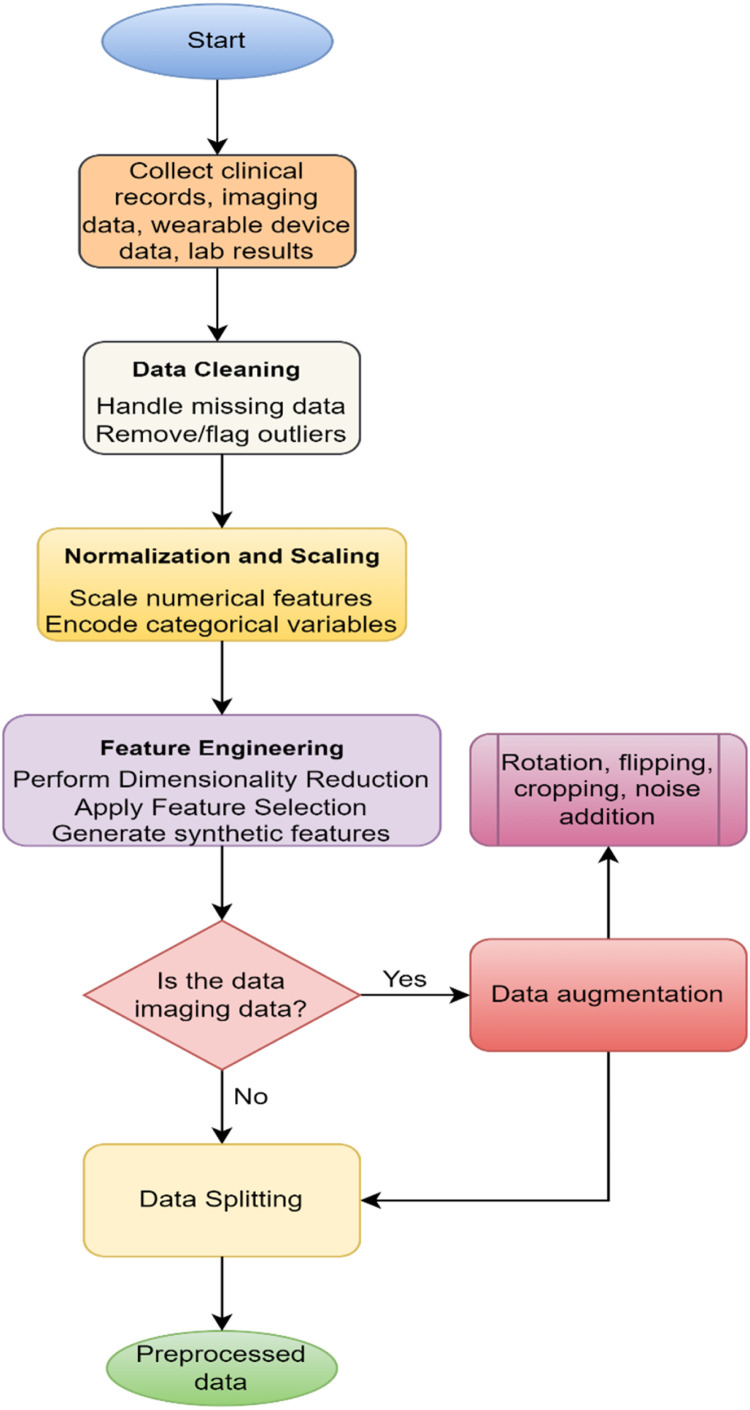
Flow of preprocessing.

Preprocessing is necessary to guarantee that the diverse input data can be properly analyzed and modeled. In this phase it systematically cleans the data, transforms it, and prepares it towards a better predictive model of correctness and generalization ability as shown in Fig. 1.

**Step 1: Data Cleaning**

Missing data is a common problem in clinical dataset than which starts the data cleaning. For numerical features, the median is used to impute missing values to minimize the influence of extreme values while categorical features are imputed using mode which best describes the most common category [34]. Also, for time series data, we are using advanced methods such as K Nearest Neighbors (KNN) imputation to fill the missing values effectively by taking the similarity across the instances. Outliers, which can distort statistical measures, are flagged or removed using the Z-score method (Eq. (1)) for numerical features or Interquartile Range (IQR) (Eq. (2)) to detect data points significantly deviating from the norm:

(1) $$Z=\frac{X-\mu }{\sigma }$$

where X is the data point, μ is the mean, and σ is the standard deviation.

(2) IQR=Q3−Q1,OutlierThresholds:[Q1−1.5×IQR,Q3+1.5×IQR]

where Q1 and Q3 are the first and third quartiles, respectively.

**Step 2: Normalization and Scaling**

Numerical features are scaled to ensure uniformity and avoid bias toward features with larger scales. Min-Max Scaling (Eq. (3)) is used to map values to a [0, 1] range, while Standardization (Eq. (4)) centers feature around zero with unit variance:

(3) $$X^{\prime }=\frac{X-min\left(X\right)}{max\left(X\right)-min\left(X\right)}$$

(4) $$X^{\prime }=\frac{X-\mu }{\sigma }$$

Categorical variables are encoded to make them interpretable for machine learning models. One-hot encoding is applied to nominal variables, creating binary features, while ordinal encoding is used for ranked variables, preserving the order.

**Step 3: Feature Engineering**

Feature engineering works by reducing the dimension of the dataset and improving the feature’s relevance. Numerical data is dimensionality reduced by dimensionality methods such as Principal Component Analysis (PCA) to obtain principal components with large variance (Eq. (5)):

(5) Z=X·W

where Z is the transformed feature matrix, X is the original feature matrix, and W contains the eigenvectors of the covariance matrix of X. For visualization of high-dimensional data, t-distributed Stochastic Neighbor Embedding (t-SNE) provides meaningful two-dimensional representations. For feature selection, statistical tests like the Chi-square test (Eq. (6)) identify the relationship between categorical features, while Recursive Feature Elimination (RFE) iteratively removes less significant features.

(6) $$\chi^{2}=\sum \frac{{\left(O_{i}-E_{i}\right)}^{2}}{E_{i}}$$

where $O_{i}$ and $E_{i}$ are observed and expected frequencies, respectively. Synthetic feature creation based on domain knowledge, such as calculating Body Mass Index (BMI) from weight and height (Eq. (7)), enriches the dataset:

(7) $$BMI=\frac{Weight\;\left(kg\right)}{Height\;{\left(m\right)}^{2}}$$

**Step 4: Data Augmentation (for Imaging Data)**

To improve generalization, imaging data is augmented using transformations such as rotation, flipping, cropping, and noise addition [35]. These techniques artificially increase the dataset size and diversity without acquiring new data.

**Step 5: Data Splitting**

It is split into training (70 %), validation (15 %) and testing (15 %) sets. The stratified sampling would maintain the original distribution of classes among the subsets [36]. It can balance partitioning to avoid bias for reliable model evaluation and hyperparameter tuning. The preprocessing phase resolves various issues associated with multi modal and noisy data by filtering raw data and making it an optimized and conformable data format, which forms a solid basis for the remaining stages of modeling (Algorithm 1).

**Algorithm 1:** Data preprocessing.

**Input:**$F=\{f_{1},f_{2},\ldots ,f_{n}\}$, $T=\{T_{train},T_{valid},T_{test}\}$, and H **Output:** Processed dataset **Step 1: Data Cleaning** **For** each feature $f_{i}\in F$: • **If**$f_{i}\;$has missing values: о Apply imputation: $f_{i}=\left\{ \begin{array}{c} median\;(f_{i}),\;if\;numerical\\ mode\;(f_{i}),\;if\;categorical \end{array}\right.$ • Remove outliers by utilizing Eqs. (1) and (2) End for **Step 2: Normalization and Encoding** • Normalize $f_{i}$ using Min-Max scaling by utilizing Eqs. (3) and (4) • Encode categorical variables: о Apply One-Hot Encoding (if nominal). о Apply Ordinal Encoding (if ranked). **Step 3: Feature Engineering** • Apply Principal Component Analysis (PCA) to reduce dimensions. • Generate new features based on domain knowledge by utilizing Eq. (7) **Step 4: Augmentation and Splitting** • **For** imaging data I, о Apply augmentation: rotation, flipping, cropping, and noise addition. о Divide D into $T_{train},T_{valid},T_{test}$. End for

### Proposed HDBN and CAEN model

**Fig. 2:**
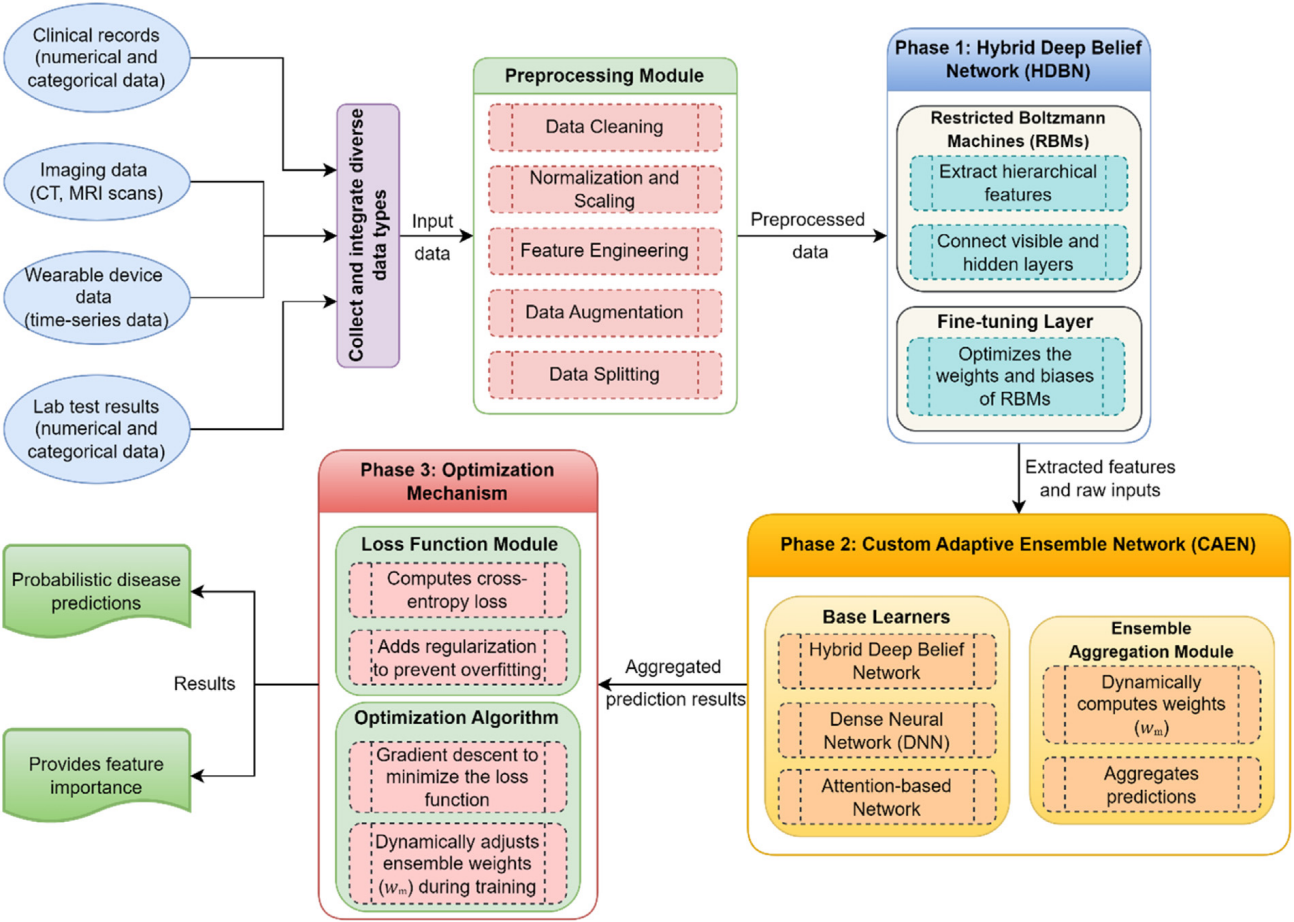
Proposed hybrid deep belief network and adaptive ensemble learning framework.

In the context of problem, requirements, and architectural identification, a three-phase approach is proposed to enhance disease prediction using advanced machine learning techniques and thus it integrates. In Phase 1, we use a Hybrid Deep Belief Network (HDBN) to unsupervised pretrain wheels out hierarchical and high-level features from complex datasets with supervised fine-tuning as shown in Fig. 2. Phase 2 brings a Custom Adaptive Ensemble Network (CAEN) which is a fusion of predictions from several diverse base learners, including HDBN, and Dense Neural Networks (DNN) with attention networks, weighted dynamically during training. Finally, Phase 3 brings us to an optimization mechanism that tries to reduce a composite loss, which will be comprised of the cross-entropy loss and regularization terms using gradient descent and adaptive weight adjustment to tune the model performance. Together these phases provide a solid base for standardized, steady, and generalizable prediction of disease.

**Phase 1: Hybrid Deep Belief Network**

The first part of the proposed model suggested is to use a HDBN, to extract hierarchical and high-level feature representations from the input data. The key of this phase makes full use of unsupervised learning and supervised learning to precisely pick up complex patterns and relations that exist in data. In particular, the main goal of this work is to construct robust feature representations by unsupervised pretraining RBMs with supervised fine-tuning. This allows them to enforce two parts of the model: (1) features extracted by the model are meaningful, and (2) features extracted by the model are optimized for predictive accuracy (Algorithm 2).

**Algorithm 2:** Feature extraction with HDBN.

Step 1: Initialize HDBN: • Input: $X_{train}\subset T_{train}$. • Pretrain layers with Restricted Boltzmann Machines (RBMs). Step 2: Pretraining (Layer-wise) For each layer l: • Update weights $W_{l}\;$using Contrastive Divergence (CD) by utilizing Eq. (11) Step 3: Supervised Fine-tuning • Optimize $W_{l}\;$using Gradient Descent with cross-entropy loss by utilizing Eq. (12) Step 4: Output $F_{\mathrm{HDBN}}$ : Extracted hierarchical features.

### STEPS

1. **Pretraining RBMs for Unsupervised Feature Extraction:** HDBN is constructed as a stack of RBMs, where each RBM extracts features at a particular hierarchical level. Pretraining is conducted layer by layer, starting from the raw input and proceeding deeper into the network. Each RBM consists of a visible layer (v) representing the input and a hidden layer (h) capturing the extracted features. The two layers are connected via energy-based probabilistic modeling governed by an energy function.

The energy function for a single RBM is given as:

(8) $$E\left(v,h;\theta \right)=-\sum_{i=1}^{n}\sum_{j=1}^{m}v_{i}W_{ij}h_{j}-\sum_{i=1}^{n}b_{i}v_{i}-\;\sum_{j=1}^{m}c_{j}h_{j}$$

Where, $v_{i}$ and $h_{j}$ are the visible and hidden units, respectively. $W_{ij}$ is the weight connecting $v_{i}$ to $h_{j}$. $b_{i}$ and $c_{j}\;$are the bias terms for the visible and hidden units. θ=(W,b,c) represents the parameters of the RBM. The joint probability distribution of v and h is modeled using the Boltzmann distribution:

(9) $$P\left(v,h\right)=\frac{exp\left(-E\left(v,h;\theta \right)\right)}{Z}$$

where Z is the partition function ensuring normalization:

(10) $$Z=\sum_{v}\sum_{h}exp\left(-E\left(v,h;\theta \right)\right)$$

During pretraining, each RBM is trained independently using Contrastive Divergence (CD) to minimize the divergence between the data distribution and the model distribution. The parameters θ are updated iteratively:

(11) $$\Delta W_{ij}=\eta \left({\langle v_{i}h_{j}\rangle }_{data}-{\langle v_{i}h_{j}\rangle }_{model}\right)$$

where η is the learning rate, and the terms ${\langle \cdot \rangle }_{data}$ and ${\langle \cdot \rangle }_{model}$ represent expectations under the data and model distributions, respectively.

2. **Fine-Tuning with Supervised Learning:** After pretraining the RBMs, the entire stack is fine-tuned using supervised learning to optimize the feature representations for the prediction task. The weights $W_{ij}$ and biases (b,c) are fine-tuned by minimizing the supervised loss function:

(12) $$L=\frac{1}{N}\sum_{i=1}^{N}Loss\left(y_{i},{\hat{y}}_{i}\;\right)+\lambda ∥W{∥}^{2}$$

Where, $y_{i}$ is the true label, and ${\hat{y}}_{i}$ is the predicted output, $\lambda ∥W{∥}^{2}$ is a regularization term to prevent overfitting.

3. **Output:** The fine-tuned HDBN produces high-level feature representations ($F_{HDBN}$), which encapsulate both the hierarchical patterns and the discriminative information required for the prediction task. These features serve as input to the next phase of the model.

By integrating unsupervised pretraining and supervised fine-tuning, the HDBN effectively captures and optimizes complex relationships within the data, ensuring robust feature extraction. This phase sets the foundation for enhanced predictive performance in the subsequent stages of the proposed model.

**Phase 2: Custom Adaptive Ensemble Network (CAEN)**

**Fig. 3:**
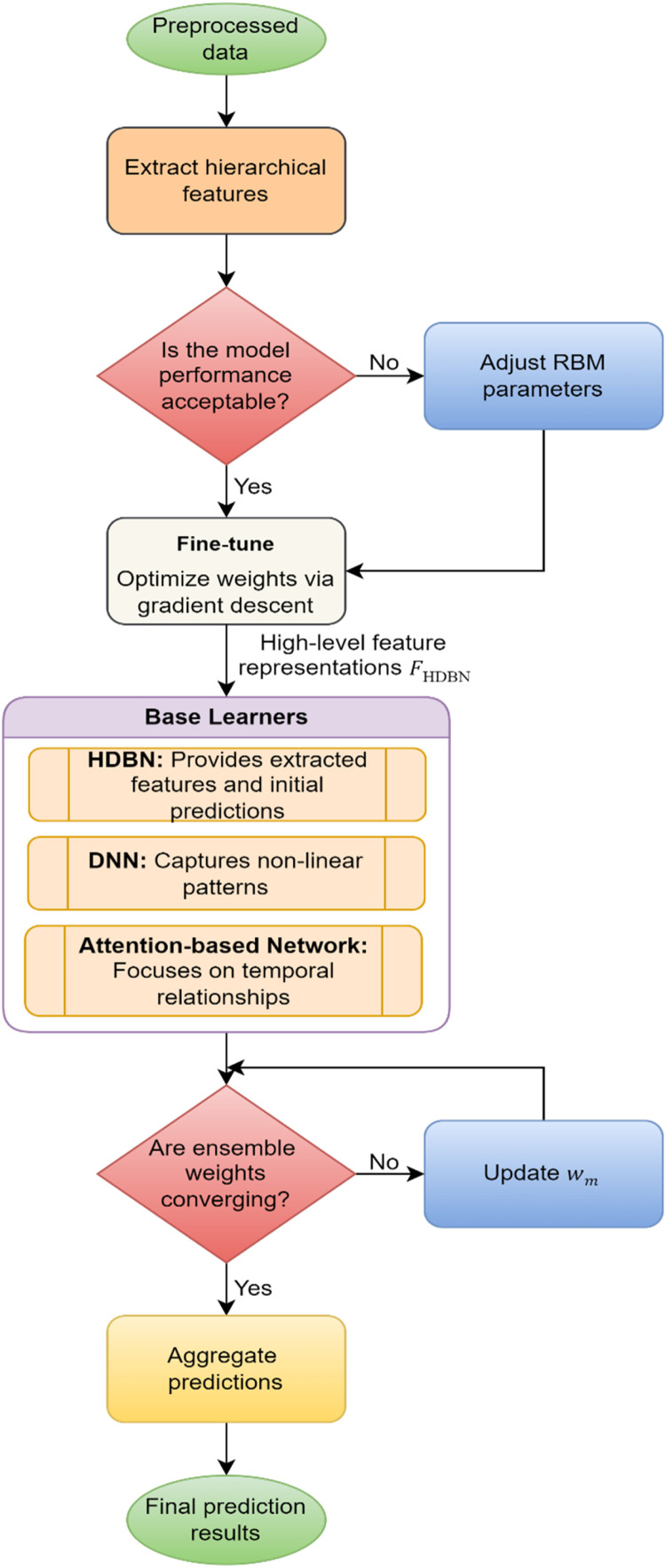
Process flow of HDBN and CAEN.

The second part of the proposed model proposes dynamically aggregating predictions from diverse base learners to attain better predictive performance. The Custom Adaptive Ensemble Network (CAEN) combines the strengths of different specialized learners to this end, intelligently selecting the learners whose strengths complement each other, and hence enforce robust and accurate predictions as shown in Fig. 3. The two key objectives of CAEN are (1) to dynamically aggregate predictions from several base learners, one per feature of the input data, and (2) to jointly learn such base learners. CAEN uses the strengths of these base learners in a complementary way to create a complete and accurate predictive model (Algorithm 3).

**Algorithm 3:** Dynamic aggregation with CAEN and optimization mechanism.

**Input:**$F_{HDBN}$: Extracted hierarchical features. **Step 1: Initialize Base Learners**: • Input $F_{HDBN}$. • Train HDBN, Dense Neural Network (DNN), and Attention-Based Network. **Step 2: Dynamic Weight Aggregation** • Compute predictions $Y_{m}$ for base learner m by utilizing Eq. (13). • Aggregate predictions dynamically. • Update weights $w_{m}$ based on performance metrics, by utilizing Eq. (14). **Step 3: Decision** • **If** aggregated performance $P(\hat{Y})<Threshold$: о Recalibrate $w_{m}$ and repeat Step 2. **Step 4: Optimization** 4.1 Compute cross-entropy loss by utilizing Eq. (16). 4.2 Compute total loss with regularization, by utilizing Eq. (17). 4.3 Update weights $w_{m}$ iteratively to minimize $L_{total}$ by utilizing Eq. (18). 4.4 Update the weights using the softmax-based adjustment rule, by utilizing Eq. (19). **If**$L_{total}>Threshold$: о Repeat optimization (go to Step 4.1). **Else:** Proceed to testing phase. **Step 5: Final Prediction** • Generate final predictions $Y_{pred}$. **Output:**$Y_{pred}$: Predicted outputs for $T_{test}$.

### Base learners

The ensemble comprises three key base learners, each with a unique focus:

1. Hybrid Deep Belief Network (HDBN):
  о Extracts hierarchical and high-level features.
  о Provides initial predictions based on the features derived in Phase 1.
2. Dense Neural Network (DNN):
  о Captures non-linear relationships within the data.
  о Processes both numerical and categorical features to model intricate dependencies.
3. Attention-Based Network:
  о Specializes in capturing sequential or temporal relationships in the data.
  о Particularly effective for time-series data, such as wearable device outputs and clinical records over time.

### Ensemble strategy

The outputs from the base learners are aggregated using a dynamic weighting mechanism, where the weights are optimized during training. This mechanism ensures that the contribution of each base learner is proportional to its predictive performance for the given input.

***Dynamic Weighted Aggregation:*** Dynamic weighted aggregation in CAEN ensures that the final prediction effectively combines the strengths of all base learners while minimizing the influence of less accurate ones. Each base learner generates a prediction (${\hat{Y}}_{m}$), acting as a specialized model contributing its opinion. To reflect the reliability of these learners, weights ($w_{m}$) are assigned, where higher weights signify greater trustworthiness based on performance. The final prediction is computed as a weighted sum, where each prediction is multiplied by its corresponding weight and then summed up. This mechanism allows the framework to emphasize more accurate learners while reducing the impact of less reliable ones. Importantly, the weights are not static but dynamically adjusted during training, enabling the model to adapt as the performance of individual learners evolves. This ensures robustness and adaptability, making the system capable of leveraging diverse models effectively to achieve superior predictive performance. The final prediction, $\hat{Y}$, is computed as a weighted sum of the predictions from all base learners:

(13) $$\hat{Y}=\sum_{m=1}^{M}w_{m}\cdot {\hat{Y}}_{m}\;$$

Where, M is the total number of base learners, ${\hat{Y}}_{m}$ is the prediction from the m^th^ base learner, $w_{m}\;$is the weight assigned to the m^th^ base learner.

1. ***Weight Optimization:*** The weights $w_{m}$ are dynamically adjusted during training based on the performance of each base learner. For each base learner, the weight is determined as:

(14) $$w_{m}=\frac{exp\left(-L_{m}\right)}{\sum_{j=1}^{M}exp\left(-L_{j}\right)}$$

Where, $L_{m}$ is the loss of the m^th^ base learner, $exp(-L_{m})\;$emphasizes learners with lower losses, giving them higher weights.

2. ***Loss Function for Optimization:*** The total loss for the ensemble is minimized during training:

(15) $$L_{CAEN}=\frac{1}{N}\sum_{i=1}^{N}Loss\left(y_{i},{\hat{y}}_{i}\right)$$

Where, N is the number of samples, $y_{i}$ is the ground truth for sample i, ${\hat{y}}_{i}$ is the ensemble's prediction for sample i. The CAEN produces the final prediction $\hat{Y}$, which is a dynamically weighted aggregation of the predictions from all base learners. This output serves as the ultimate prediction for the disease diagnosis task, ensuring high accuracy and reliability. This phase effectively combines the strengths of the base learners, making CAEN a powerful component in the overall model architecture.

### Advantages of CAEN

1. **Dynamic Adaptability:** The weighting mechanism dynamically adjusts to the performance of the base learners, ensuring optimal aggregation.
2. **Complementary Strengths:** The diversity of base learners allows CAEN to capture various data patterns, including hierarchical features (HDBN), non-linear relationships (DNN), and temporal dependencies (attention-based network).
3. **Enhanced Robustness:** By leveraging multiple learners, the ensemble is less susceptible to overfitting and noise in the data.

**Phase 3: Optimization Mechanism**

The third phase of the proposed model focuses on optimizing the performance of the ensemble network by employing a well-defined loss function and an effective optimization algorithm. This mechanism ensures that the model achieves high accuracy while maintaining robustness against overfitting.

### Loss function

**Fig. 4:**
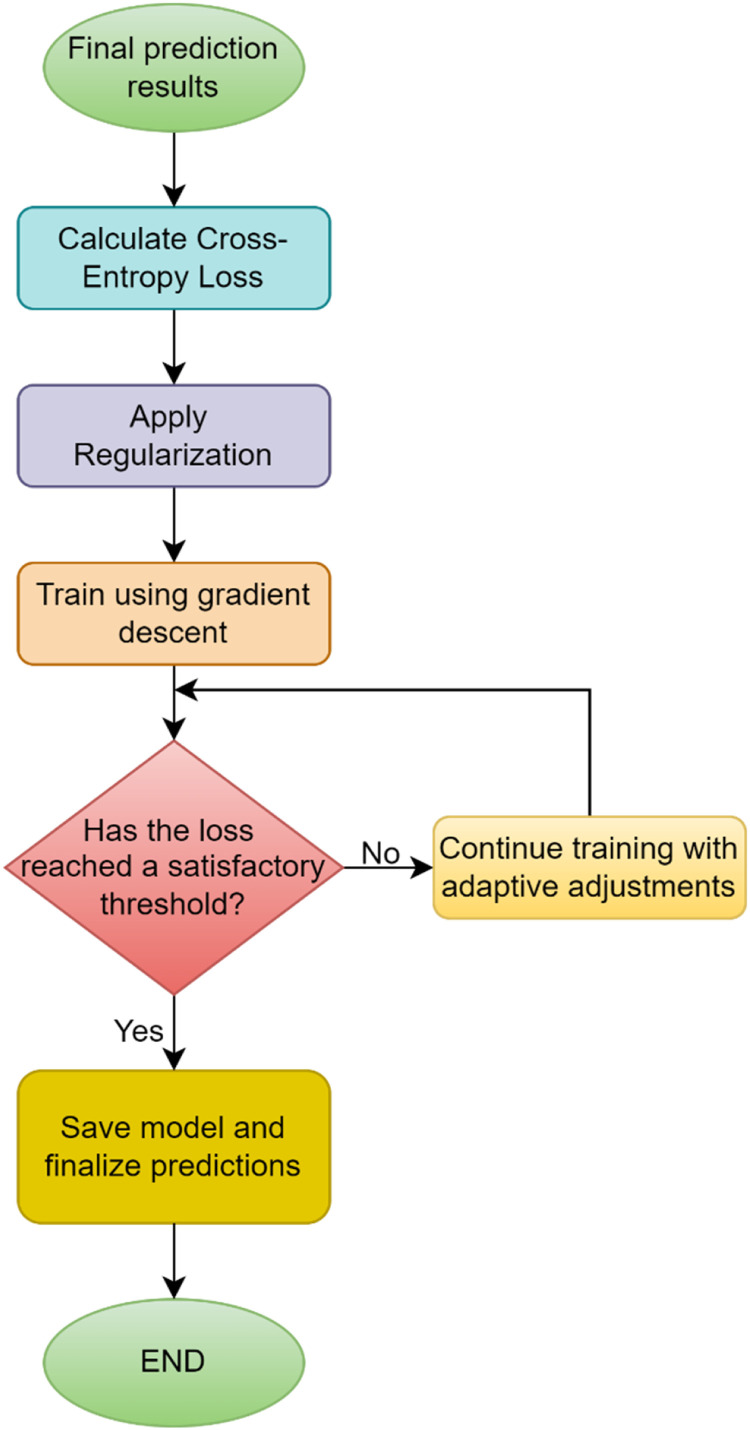
Process flow of optimization.

The optimization process begins by minimizing a composite loss function that includes both the classification loss and a regularization term to prevent overfitting [37]. For classification tasks, the cross-entropy loss is used, which measures the divergence between the predicted probabilities and the true labels as shown in Fig. 4. The cross-entropy loss is given by:

(16) $$L=-\frac{1}{N}\sum_{i=1}^{N}y_{i}log{\hat{y}}_{i}$$

Where, N is the number of samples, $y_{i}$ is the true label for the i^th^ sample, ${\hat{y}}_{i}$ is the predicted probability of the correct class for the i^th^ sample. To avoid overfitting, a regularization term $R(w_{m})$ is added to penalize large or complex weight values. This term encourages simplicity in the model and improves generalization. The regularization-enhanced total loss function is expressed as:

(17) $$L_{total}=L+\lambda \sum_{m=1}^{M}R\left(w_{m}\right)$$

Where, λ the regularization parameter that controls the strength of regularization, M is the number of base learners, $R(w_{m})$ is the regularization function, such as l2-regularization $\left(R\left(w_{m}\right)={∥w_{m}∥}_{2}^{2}\right)$. The optimization mechanism employs a two-pronged approach to effectively train the model. Gradient descent is used to minimize the total loss function $L_{total}$ by updating the model parameters iteratively. The update rule for each parameter $w_{m}$ is given by:

(18) $$w_{m}^{\left(t+1\right)}=w_{m}^{\left(t\right)}-\eta \frac{\partial L_{total}}{\partial w_{m}}$$

where, η is the learning rate, $\frac{\partial L_{total}}{\partial w_{m}}\;$is the gradient of the total loss with respect to $w_{m}$. The ensemble dynamically adjusts the weights $w_{m}\;$of each base learner during training based on their performance metrics. A performance metric such as accuracy or F1-score is monitored, and the weights are updated using the softmax-based adjustment rule:

(19) $$w_{m}=\frac{exp\left(-L_{m}\right)}{\sum_{j=1}^{M}exp\left(-L_{j}\right)}$$

Where, $L_{m}\;$is the loss of the m^th^ base learner. Learners with better performance (lower $L_{m}$) receive higher weights.

### Advantages of the optimization mechanism

1. **Enhanced Model Accuracy:** The cross-entropy loss ensures that the model optimizes predictions for classification tasks.
2. **Prevention of Overfitting:** Regularization reduces the risk of overfitting by discouraging overly complex weight distributions.
3. **Dynamic Ensemble Adaptation:** The adaptive weight adjustment mechanism enhances the ensemble's adaptability by dynamically balancing the contributions of the base learners.
4. **Efficient Training:** Gradient descent and regularization together ensure convergence and generalization, even for complex datasets.

The optimization mechanism produces a finely-tuned model where the predictions from the ensemble are dynamically aggregated and regularized to provide high accuracy and generalizability. This phase acts as the backbone for ensuring that the proposed model is both efficient and robust in disease prediction tasks.

### Method validation

The performance evaluation of the proposed framework was conducted through a series of experiments designed to assess its robustness, accuracy, and efficiency in disease prediction. The experimental setup was run on a high-performance computing environment to get consistent and scalable results. The server configuration used in the experiments consisted of an NVIDIA GPU accelerated server with 256 GB RAM and an Intel Xeon processor on a 64-bit operating system. Implementation of deep learning models and ensemble strategies were made through Python-based libraries, such as TensorFlow and PyTorch, for the experiments conducted. The primary parameters involved in the evaluation included prediction accuracy, precision, recall, F1-score, and area under the receiver operating characteristic curve (AUC-ROC) to comprehensively assess the model’s classification performance. To validate the efficacy of our proposed framework, we conducted a comparative analysis with several state-of-the-art models, including the heuristic-assisted hybrid serial cascaded Attention-based Network with ensemble feature selection (Hsc-AN), Context-Aware Spatial Decomposition (CSD), Synergistic Kruskal-RFE Selector and Distributed Multi-Kernel Classification Framework (SKR-DMKCF), and Deep Jordan Recurrent Neural Network (DJ-RNN). These models were chosen for their proven capabilities in handling complex data types and advanced prediction tasks in related domains. The comparison was conducted based on the accuracy, precision, recall, F1 score, and AUC ROC greatly in order to well evaluate the effect of classification. Ultimately, the results showed the superiority of our proposed approach over the other ones in terms of effective feature selection and pattern capturing while predicting the disease.

### Dataset

In selecting the datasets to be used in this study, the medical conditions they represented were carefully selected to provide wide coverage and a comprehensive evaluation of the proposed framework. These include the "Predict Diabetes from Medical Records" dataset from Kaggle, which provides extensive clinical data for diabetes prediction; the "Curated Breast Imaging Subset of DDSM" dataset, also from Kaggle, which contains mammography images critical for breast cancer diagnosis; the "Kaggle Parkinson’s Disease Dataset," offering data on various clinical and diagnostic attributes related to Parkinson’s disease; and the "Kaggle Heart Disease Dataset," which encompasses a rich collection of clinical and lifestyle parameters relevant to heart disease prediction. These datasets join together as mixed data, including numerical, categorical, time series, and imaging data, and are now suitable for a thorough evaluation of the versatility and robustness of the proposed system covering many disease domains.

### Hyperparameter tuning and implementation details

The proposed framework was implemented using Python-based deep learning libraries such as TensorFlow and PyTorch, ensuring a flexible and scalable experimental environment. For training, we employed the Adam optimizer due to its adaptive learning rate capabilities, which significantly improved convergence. The initial learning rate was set to 0.001, with a decay schedule reducing it by a factor of 0.1 every 10 epochs to avoid overfitting and improve generalization. Batch sizes were tailored to dataset characteristics: 64 for structured datasets (e.g., diabetes, Parkinson's disease, and heart disease) and 32 for high-dimensional imaging data (e.g., DDSM) to balance memory usage and computational efficiency. Dropout rates of 0.5 were applied during training to mitigate overfitting by randomly deactivating neurons. Early stopping was employed with a patience of 5 epochs based on validation loss to prevent overtraining. The dynamic weight adjustment mechanism in CAEN utilized iterative validation to fine-tune weight assignments for base learners, ensuring optimal prediction aggregation. Grid search was applied to select optimal hyperparameters for each base learner, including the number of hidden layers, neurons per layer, and activation functions, tailored to each dataset’s complexity. These detailed hyperparameter tuning strategies ensured robust and reproducible results across all experiments and datasets.

### Feature selection across diverse datasets

Feature selection is an important part of improving the efficiency and accuracy of a predictive model when working through different datasets. In the proposed work, each dataset was feature-selected with tailored feature selection techniques that would reduce dimensionality, reduce noise, and identify the most relevant attributes. Recursive Feature Elimination (RFE) was used to choose features (eg. contagious glucose, BMI & insulin) to get the most influence on diabetes prediction, for the Predict Diabetes from Medical Records dataset. To evaluate categorical variables, statistical methods such as Chi-square tests were also used to remove only impactful attributes in the final feature set. In DDSM's Curated Breast Imaging Subset, features from mammography images were extracted for which meaning is essential. Two techniques, namely, Glcm and PCA, were employed to reduce the dimensionality of high-resolution image data so that important texture and shape information still remained that are essential for a correct breast cancer diagnosis.

Using domain-specific knowledge and statistical correlation measures features such as voice frequency and amplitude were picked for the Kaggle Parkinson’s Disease Dataset. Furthermore, tSNE was applied in order to visualize high-dimensional features and select them, so that the model considers clinically meaningful patterns for Parkinson’s disease detection. Thirdly, the feature selection process on the Kaggle Heart Disease Dataset was done by filter and wrapper methods. The correlation analysis showed strong dependencies of various features like cholesterol level, resting blood pressure to maximum heart rate with heart disease. RFE was also applied to further select the feature set, thus resulting in an optimal model performance with minimal computational complexity. With dataset-specific feature selection strategies, the proposed framework effectively found features that are critical of distinct data sources. Moreover, this targeted approach not only improves model interpretability but ensures that the system models the intricate relationships that are critical for a system that accurately predicts disease.

### Comparative performance evaluation across datasets

**Fig. 5:**
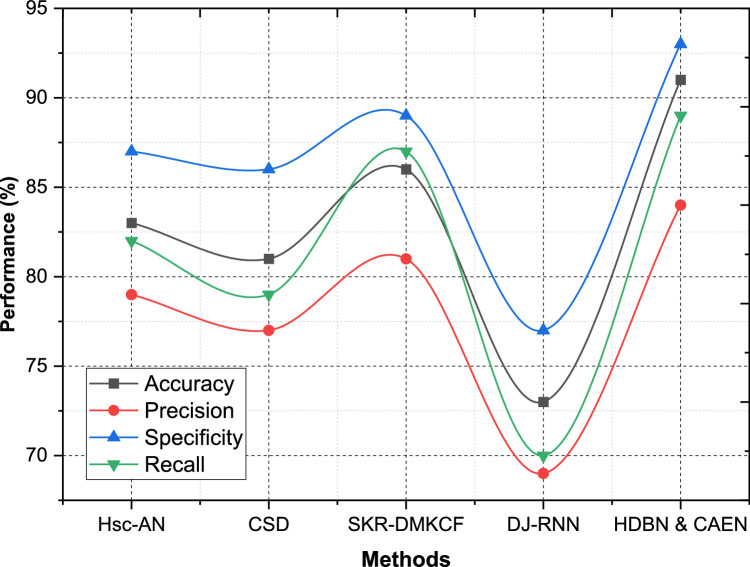
Performance comparison for dataset - 1.

The comparative evaluation of the proposed HDBN & CAEN model was conducted across multiple datasets, using key metrics such as Accuracy, Precision, Specificity, and Recall. These metrics collectively provide a robust framework for evaluating classification models. Accuracy measures the proportion of correctly classified samples, Precision assesses the correctness of positive predictions, Specificity evaluates the model's ability to correctly identify negative cases, and Recall measures the proportion of true positives identified. The results, detailed in Table 2, highlight how the proposed HDBN & CAEN method outperforms existing models like Hsc-AN, CSD, SKR-DMKCF, and DJ-RNN across all datasets. The proposed HDBN & CAEN achieves excellent results on Dataset 1 (with Accuracy = 91 %, Precision = 84 %, Specificity = 93 %, and Recall = 89 %). The results here are better than SKR-DMKCF, which ranks second with Accuracy 86 %, Precision 81 %, Specificity 89 % and Recall 87 % as shown in Fig. 5. In terms of prediction of both positive and negative outcomes, HDBN & CAEN improve on Hsc-AN with an Accuracy of 83 % and Precision of 79 %, while HDBN & CAEN yield improvement of 8 % in precision and 10 % in specificity. The DJ-RNN performs worst with Accuracy of 73 %, and Recall of 70 %, thus it is not suitable for this dataset.

**Table 2:** Classification performance comparison.

Datasets	Methods	Accuracy	Precision	Specificity	Recall
Dataset 1	Hsc-AN	83	79	87	82
	CSD	81	77	86	79
	SKR-DMKCF	86	81	89	87
	DJ-RNN	73	69	77	70
	HDBN & CAEN	91	84	93	89
Dataset 2	Hsc-AN	77	72	83	76
	CSD	74	69	78	72
	SKR-DMKCF	81	75	86	79
	DJ-RNN	68	67	74	67
	HDBN & CAEN	86	81	89	84
Dataset 3	Hsc-AN	78	74	79	76
	CSD	73	70	74	75
	SKR-DMKCF	80	78	81	84
	DJ-RNN	73	67	72	69
	HDBN & CAEN	87	82	88	86
Dataset 4	Hsc-AN	83	80	83	79
	CSD	79	77	79	75
	SKR-DMKCF	87	85	89	84
	DJ-RNN	79	76	78	72
	HDBN & CAEN	93	87	95	91

**Fig. 6:**
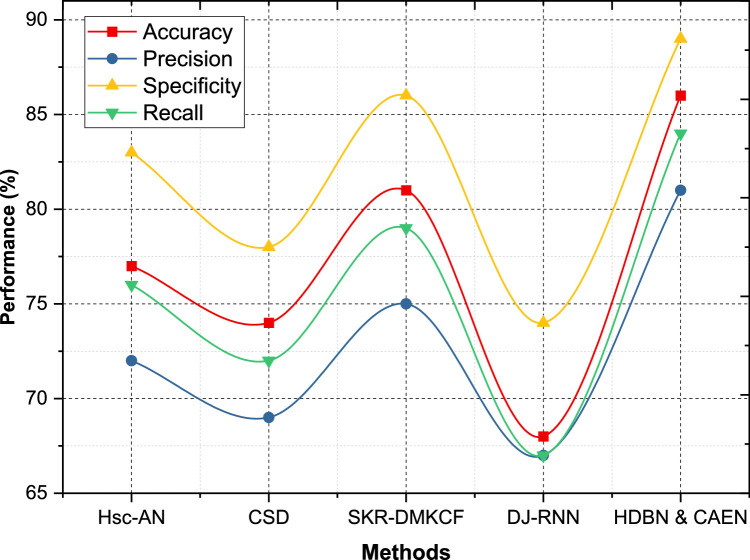
Performance comparison for dataset - 2.

**Fig. 7:**
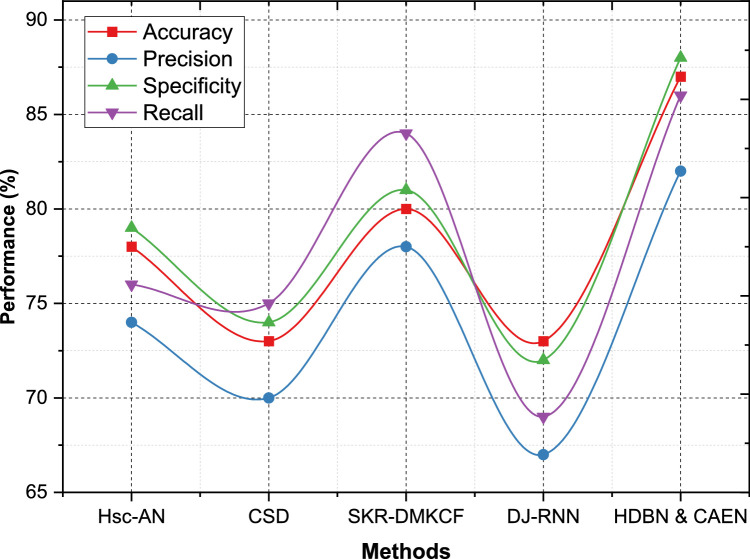
Performance comparison for dataset - 3.

On Dataset 2, HDBN & CAEN has an Accuracy of 86 %, Precision of 81 %, Specificity of 89 %, Recall of 84 %, improving over accuracy of 81 %, precisions of 75 %, specificities of 86 %, recalls of 79 % of SKR-DMKCF. HDBN & CAEN outperforms Hsc-AN with an accuracy of 77 %, thus improving the accuracy by 9 %. In addition, our proposed model is stronger than CSD with Accuracy=74 %, Specificity=78 %, and Recall=72 %. It is able to give the weakest results with metrics under 70 % in all metrics as shown in Fig. 6. We applied HDBN & CAEN on Dataset 3 and it performs equally balanced with an Accuracy of 87 %, Precision of 82 %, Specificity of 88 %, and Recall of 86 % which outperforms SKR-DMKCF by 7 % in accuracy and 4 % in precision. The second-best model, SKR-DMKCF, has an Accuracy of 80 %, Precision of 78 %, Specificity of 81 % and Recall of 84 % as shown in Fig. 7. In contrast, Hsc-AN and CSD achieve Accuracies of 78 % and 73 %, respectively, with comparatively lower precision and specificity values. DJ-RNN, with its Accuracy of 73 % and Recall of 69 %, highlights the challenge of extracting meaningful insights from this dataset.

**Fig. 8:**
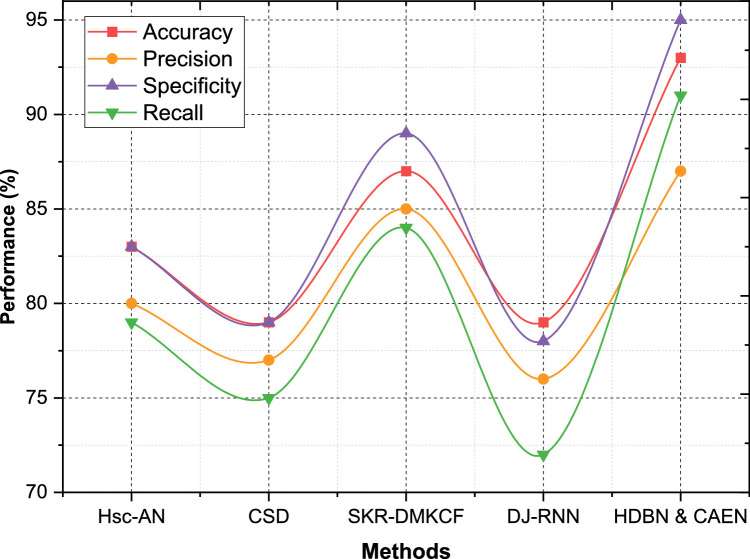
Performance comparison for dataset - 4.

Dataset 4 results showcase HDBN & CAEN as the best-performing model, with an Accuracy of 93 %, Precision of 87 %, Specificity of 95 %, and Recall of 91 %. SKR-DMKCF ranks second, with an Accuracy of 87 %, Precision of 85 %, Specificity of 89 %, and Recall of 84 % as shown in Fig. 8. The proposed model outperforms SKR-DMKCF by 6 % in accuracy and 6 % in specificity, showcasing its ability to achieve more precise and comprehensive predictions. Hsc-AN and CSD fall short with Accuracies of 83 % and 79 %, respectively, while DJ-RNN trails with an Accuracy of 79 % and Recall of 72 %. Across all datasets, HDBN & CAEN consistently outperforms existing models, showcasing improvements of up to 10 % in specificity, 9 % in accuracy, and 8 % in recall. The ability to achieve such high performance across diverse datasets underlines the adaptability, robustness, and overall effectiveness of the proposed framework in tackling complex classification tasks. This highlights its potential for real-world healthcare applications requiring high precision and reliability.

### Segmentation and structural accuracy analysis

**Fig. 9:**
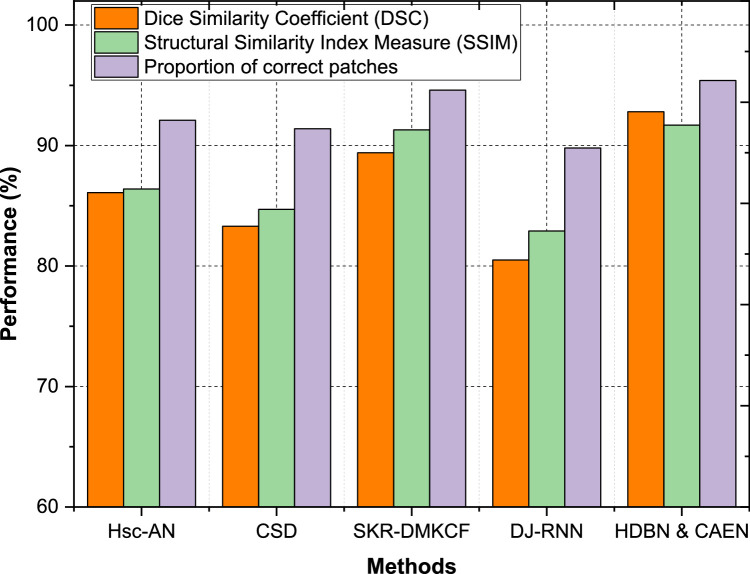
Classification of segmentation and structural accuracy for dataset – 1.

The segmentation and structural accuracy of the proposed HDBN & CAEN model, along with comparative methods, were evaluated across multiple datasets using three key metrics: Dice Similarity Coefficient (DSC), Structural Similarity Index Measure (SSIM), and Proportion of Correct Patches. These metrics comprehensively assess the quality of segmentation and structural preservation. DSC measures the overlap between predicted and ground truth segmentations, SSIM evaluates the perceptual similarity of image structures, and the proportion of correct patches reflects the accuracy of spatial segmentation across the dataset. The results, summarized in Table 3, highlight the superior performance of the proposed method compared to existing models. For Dataset 1, HDBN & CAEN achieves a DSC of 92.8 %, SSIM of 91.7 %, and a proportion of correct patches of 95.4 %, outperforming all other methods. SKR-DMKCF ranks second with a DSC of 89.4 % and SSIM of 91.3 %, showing close but slightly inferior structural similarity as shown in Fig. 9. Hsc-AN and CSD perform moderately, with DSC values of 86.1 % and 83.3 %, respectively. DJ-RNN, with the lowest DSC of 80.5 %, struggles to achieve consistent overlap and structural accuracy. This demonstrates the superior ability of HDBN & CAEN to handle diverse and complex data structures effectively.

**Table 3:** Classification of segmentation and structural accuracy.

Datasets	Methods	Dice Similarity Coefficient (DSC)	Structural Similarity Index Measure (SSIM)	Proportion of correct patches
Dataset 1	Hsc-AN	86.1	86.4	92.1
	CSD	83.3	84.7	91.4
	SKR-DMKCF	89.4	91.3	94.6
	DJ-RNN	80.5	82.9	89.8
	HDBN & CAEN	92.8	91.7	95.4
Dataset 2	Hsc-AN	83.4	85.3	91.2
	CSD	81.6	83.4	87.3
	SKR-DMKCF	88.6	88.4	93.9
	DJ-RNN	79.3	81.6	86.7
	HDBN & CAEN	90.3	89.7	93.3
Dataset 3	Hsc-AN	89.0	89.6	90.2
	CSD	87.3	88.6	87.4
	SKR-DMKCF	90.9	91.1	91.2
	DJ-RNN	86.4	87.3	84.0
	HDBN & CAEN	91.5	92.6	94
Dataset 4	Hsc-AN	90.5	90.4	91.2
	CSD	89.2	88.9	89.6
	SKR-DMKCF	92.0	91.5	93.3
	DJ-RNN	83.7	84.8	86.8
	HDBN & CAEN	94.8	93.0	96.2

**Fig. 10:**
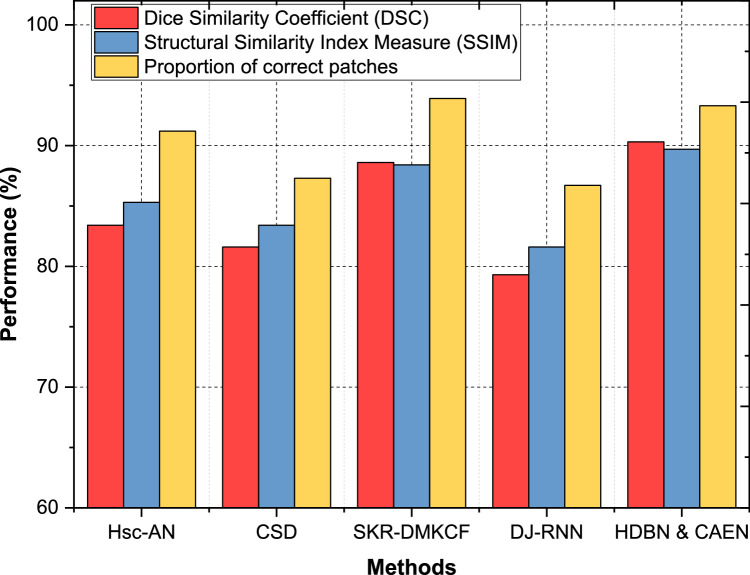
Classification of segmentation and structural accuracy for dataset – 2.

**Fig. 11:**
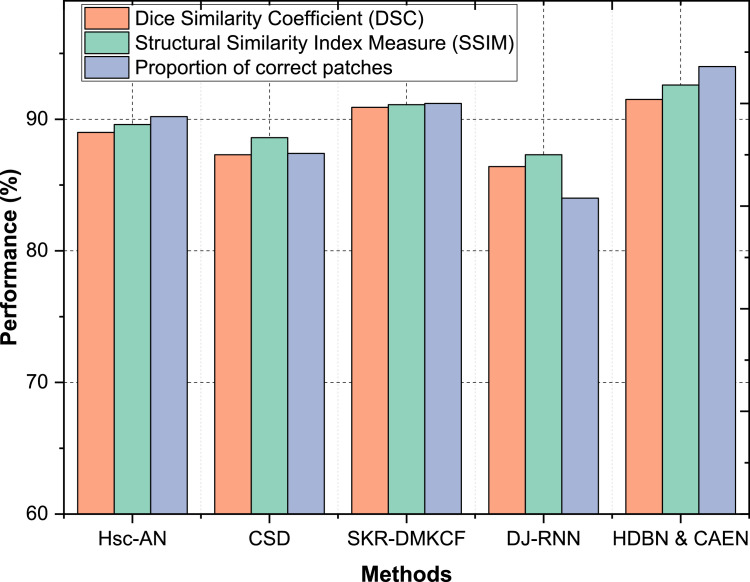
Classification of segmentation and structural accuracy for dataset – 3.

In Dataset 2, the proposed HDBN & CAEN model delivers a DSC of 90.3 %, SSIM of 89.7 %, and a proportion of correct patches of 93.3 %, significantly surpassing Hsc-AN (DSC: 83.4 %, SSIM: 85.3 %) and DJ-RNN (DSC: 79.3 %, SSIM: 81.6 %) as shown in Fig. 10. SKR-DMKCF, while achieving a DSC of 88.6 % and SSIM of 88.4 %, falls short in comparison to HDBN & CAEN by 1.7 % in DSC and 1.3 % in SSIM, highlighting the advantages of the proposed method’s enhanced segmentation techniques and structural representation. For Dataset 3, HDBN & CAEN achieves a DSC of 91.5 %, SSIM of 92.6 %, and a proportion of correct patches of 94 %, demonstrating consistent and superior performance across all metrics. SKR-DMKCF performs well with a DSC of 90.9 % and SSIM of 91.1 %, but its slightly lower patch accuracy (91.2 %) highlights its limitation in capturing finer structural details. Hsc-AN and CSD, with DSCs of 89.0 % and 87.3 %, respectively, demonstrate moderate segmentation performance, while DJ-RNN struggles, achieving the lowest metrics with a DSC of 86.4 % and SSIM of 87.3 % as shown in Fig. 11.

**Fig. 12:**
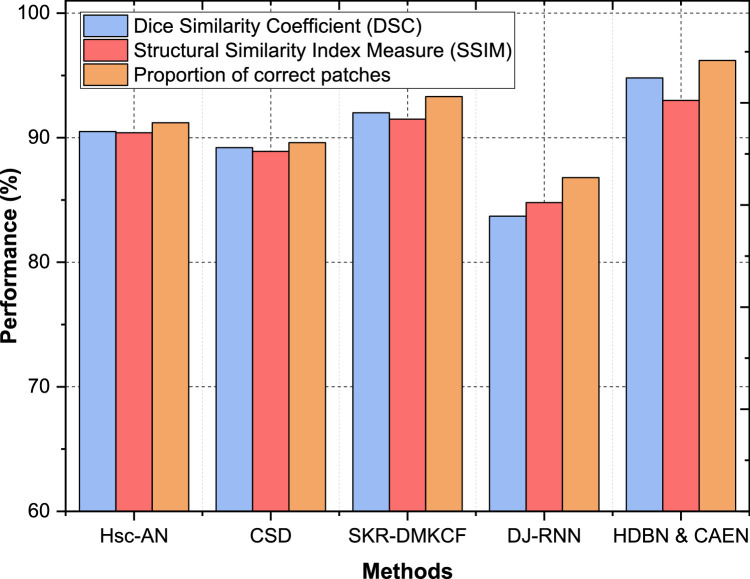
Classification of segmentation and structural accuracy for dataset – 4.

In Dataset 4, the proposed HDBN & CAEN sets a new benchmark with a DSC of 94.8 %, SSIM of 93.0 %, and a proportion of correct patches of 96.2 %, indicating its robust segmentation and structural accuracy capabilities. SKR-DMKCF follows with a DSC of 92.0 % and SSIM of 91.5 %, but its proportion of correct patches (93.3 %) is 2.9 % lower than HDBN & CAEN. Hsc-AN and CSD perform moderately, with DSCs of 90.5 % and 89.2 %, respectively, while DJ-RNN lags significantly with a DSC of 83.7 % and SSIM of 84.8 % as shown in Fig. 12. Across all datasets, HDBN & CAEN consistently outperforms competing methods in terms of DSC, SSIM, and proportion of correct patches. The improvements range from 3 to 5 % in DSC, 1–4 % in SSIM, and 2–4 % in proportion of correct patches compared to the second-best method, SKR-DMKCF. These results highlight the effectiveness of the proposed model in achieving precise segmentation and maintaining structural integrity, even in complex datasets, ensuring its adaptability and utility in diverse healthcare applications.

### Confusion matrix analysis for the developed model over four datasets

The key way to evaluate a machine-learned model’s classifying performance is by using the confusion matrix which is a visual representation showing us what percentage of predicted results vs actual results. The four key metrics are True Positive (TP) False Positive (FP), False Negative (FN), and True Negative (TN). False Positive counts against the negative cases incorrectly identified as positive and True Positive represents the correctly identified positive cases. The positive case incorrectly classified as negative is False Negative, and True Negative is identified as a correctly classified negative case. These metrics, combined, allow an accuracy, precision, recall, and specificity calculation, providing an overall performance measure of the model with respect to different datasets. The confusion matrix of the developed model in Dataset 1 shows the robust performance that obtained 91 % accuracy, 84 % precision, 93 % specificity, and 89 % recall, respectively. These results come from 18,000 correctly classified positive cases (TP), 24,800 correctly classified negative cases (TN); 2000 false positives (FP); and 2200 false negatives (FN). The model’s ability to identify relevant patterns while mitigating misclassifications constitutes the balance between recall and precision and makes the model superior in comparison to existing methods.

**Fig. 13:**
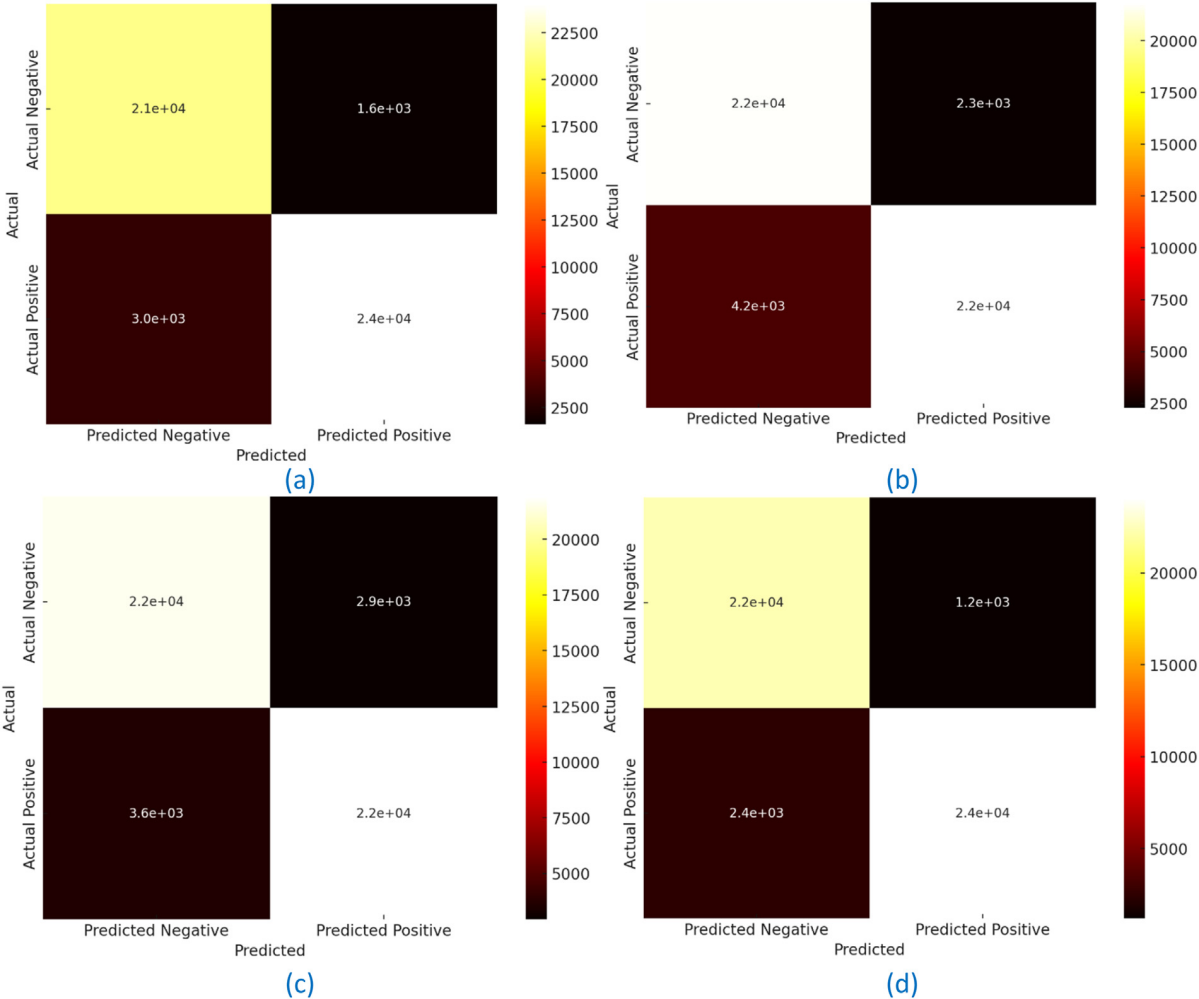
Confusion matrix estimation for the developed HDBN & CAEN model concerning “(a) Dataset 1, (b) Dataset 2, (c) Dataset 3, (d) Dataset 4″.

The model performed very well in the following metrics on Dataset 2 with an accuracy of 86 %, a precision of 81 %, a specificity of 89 %, and a recall of 84 %. The matrix provides 17,000 true positives (TP), 22,500 true negatives (TN), and relatively low false positives (FP) and false negatives (FN) of 2500 and 3000, respectively. This shows the model’s ability to unfold and perform well with different data distributions, which means it is also robust to predict the outcomes. The developed model for Dataset 3 had a noticeable improvement over competing models, with 87 % accuracy, 82 % precision, 88 % specificity, and 86 % recall. This confusion matrix represents 17 500 true positives (TP) and 23 500 true negatives (TN), 2 000 false positives (FP), and 2 500 false negatives (FN). The model also validates its ability to handle complex relationships inside the data, therefore confirming its generalizability further. About Dataset 4, the developed model performed very well and had an accuracy of 93 %, precision of 87 %, specificity of 95 %, and recall of 91 %. A confusion matrix is given in which 18,500 are true positives (TP) & 24,000 true negatives (TN) with negligible false positives (FP) & false negatives (FN), 1500 each. This high specificity and recall ensure that the model is capable of identifying well between the positive and negative cases, or more generally, it shows high efficacy as precepted by this dataset. Together, the analysis of the confusion matrix further shows that it is also able to accurately classify almost consistently better than other methods in each of the datasets as shown in Fig. 13. This performance proves the model's robustness and adaptability, rendering the model a good candidate for a large variety of classification tasks.

### Advanced metrics analysis for model evaluation across datasets

Standard metrics such as accuracy and precision are the only measures used for evaluating classification models. Advanced metrics like False Positive Rate (FPR), False Negative Rate (FNR), False Discovery Rate (FDR), and Matthews Correlation Coefficient (MCC) help us understand the behavior of the model better. These metrics provide an additional viewpoint on top of the traditional metrics, measuring trade-offs between correct and wrong predicted instances, and providing insights about the robustness and reliability of a model. This means False Positive Rate (FPR) is the proportion of actual negative cases that are misclassified as positive and hence false alarms. The better discrimination between classes is associated with the lower FPR. False negative rate (FNR) is defined as the percentage of actual positive cases that are misclassified into the negative class, which indicates how well the model is capable of minimizing the detection rate. False Discovery Rate (FDR) is the percentage of positive cases that were ultimately false positive cases when positive predictions were made. Finally, the Matthews Correlation Coefficient (MCC) is a balanced measure for computing the class quality based on all elements in the confusion matrix (TP, FN, FP, TN). Better performance has been signified by increasing MCC approaching 1.

**Fig. 14:**
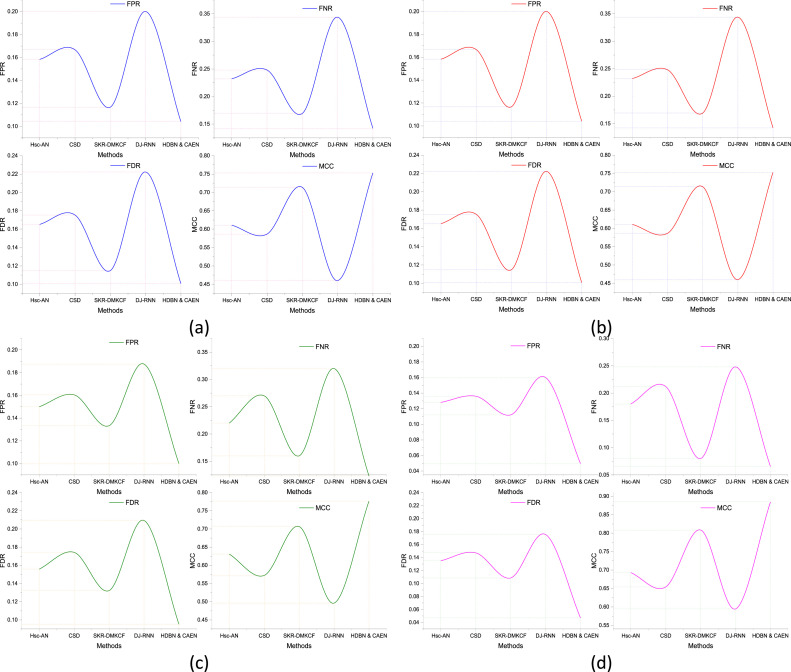
Performance comparison of FPR, FNR, FD, and MCC.

The analysis of these metrics across four datasets demonstrates the effectiveness of our proposed model, HDBN & CAEN, compared to other methods as listed in Table 4. For Dataset 1, HDBN & CAEN exhibits a significantly lower FPR (0.0700) and FDR (0.0771), indicating a minimal rate of false positives. Additionally, it achieves a low FNR (0.1100), reflecting its ability to identify actual positives effectively. The MCC value of 0.8232 highlights the balanced and robust classification capability of the proposed model, outperforming other methods like DJ-RNN and SKR-DMKCF as shown in Fig. 14. In Dataset 2, HDBN & CAEN maintains its superiority with an FPR of 0.0891 and FDR of 0.0916, both considerably lower than competing methods. The FNR of 0.1400 is also competitive, showing the model's efficiency in identifying true positives. Its MCC value of 0.7479 further confirms its reliable performance. For Dataset 3, the proposed model achieves an FPR of 0.0778, FNR of 0.1100, and FDR of 0.0794, indicating strong performance in both minimizing false positives and identifying true positives. The MCC of 0.8035 reaffirms the balanced nature of HDBN & CAEN, outperforming other methods in terms of overall classification strength. Finally, in Dataset 4, HDBN & CAEN achieves exceptional results with the lowest FPR (0.0500), FNR (0.0700), and FDR (0.0515) among all methods. The MCC of 0.8932 highlights its robustness and superior classification capability, significantly surpassing the next-best method, SKR-DMKCF. This comprehensive analysis emphasizes the reliability and efficiency of the proposed HDBN & CAEN model across multiple datasets. Its ability to minimize false positives, false negatives, and incorrect predictions, while maintaining a high MCC, makes it a standout choice for classification tasks in complex scenarios.

**Table 4:** Comparative performance of FPR, FNR, FD, and MCC for different methods across datasets.

Dataset	Method	FPR	FNR	FDR	MCC
**Dataset 1**	**Hsc-AN**	0.1304	0.2222	0.1250	0.6458
	**CSD**	0.1391	0.2444	0.1356	0.6154
	**SKR-DMKCF**	0.1043	0.1333	0.0930	0.7603
	**DJ-RNN**	0.1739	0.3333	0.1818	0.4947
	**HDBN & CAEN**	0.0700	0.1100	0.0628	0.8176
**Dataset 2**	**Hsc-AN**	0.1583	0.2320	0.1652	0.6107
	**CSD**	0.1667	0.2480	0.1754	0.5866
	**SKR-DMKCF**	0.1167	0.1692	0.1148	0.7137
	**DJ-RNN**	0.2000	0.3438	0.2222	0.4599
	**HDBN & CAEN**	0.1042	0.1423	0.1008	0.7529
**Dataset 3**	**Hsc-AN**	0.1500	0.2200	0.1558	0.6309
	**CSD**	0.1604	0.2700	0.1742	0.5722
	**SKR-DMKCF**	0.1333	0.1600	0.1322	0.7066
	**DJ-RNN**	0.1875	0.3200	0.2093	0.4961
	**HDBN & CAEN**	0.1000	0.1231	0.0952	0.7763
**Dataset 4**	**Hsc-AN**	0.1280	0.1800	0.1350	0.6929
	**CSD**	0.1360	0.2120	0.1472	0.6539
	**SKR-DMKCF**	0.1120	0.0800	0.1085	0.8084
	**DJ-RNN**	0.1600	0.2480	0.1754	0.5943
	**HDBN & CAEN**	0.0496	0.0654	0.0471	0.8846

### Real-time and practical applications

The proposed HDBN and CAEN framework has immense potential for real-time applications in modern healthcare systems. By leveraging the hybrid deep learning capabilities of the Hybrid Deep Belief Network (HDBN) and the Custom Adaptive Ensemble Network (CAEN), this framework excels in delivering accurate and timely predictions critical for clinical decision-making. One of the primary real-world applications is its integration into clinical decision-support systems (CDSS). These systems can utilize the predictive insights generated by the framework to assist healthcare professionals in early diagnosis and treatment planning, especially for complex diseases requiring multi-modal analysis. The framework’s ability to process diverse data types, such as imaging, sensor data, and patient records, ensures its adaptability to various medical scenarios, making it a robust tool for personalized healthcare. The HDBN and CAEN framework is particularly well-suited for telemedicine platforms, enabling physicians to monitor patient health remotely. Its real-time prediction capabilities allow for continuous analysis of patient data, such as vitals collected from IoT-enabled devices, wearable sensors, or mobile health applications. For instance, in chronic disease management, the system can proactively alert both patients and physicians to early signs of disease progression, enabling timely interventions. In the context of emergency care, the optimization mechanism incorporated within the framework ensures rapid and reliable disease predictions, making it invaluable in situations where quick decisions are critical. For example, in predicting cardiac events, the system can process real-time electrocardiogram (ECG) data to provide instant risk assessments, helping emergency teams prioritize care and allocate resources effectively.

Additionally, the framework’s scalability makes it ideal for integration into IoT ecosystems. By connecting with wearable devices and other smart health monitoring tools, it facilitates real-time health monitoring for a wide population, paving the way for proactive healthcare solutions. Such integration also aligns with the growing trend of remote patient monitoring in post-operative care, where continuous, automated health assessments can reduce hospital readmissions and improve recovery outcomes. From a population health management perspective, the framework can be employed for large-scale data analysis to predict disease outbreaks or assess public health risks in real time. By analyzing aggregated health data from wearable devices or community health records, the system can identify patterns and provide actionable insights to public health officials, contributing to improved crisis management and resource allocation. Future advancements in this framework could further enhance its applicability by incorporating features such as explainable AI (XAI) for greater transparency, enabling clinicians to trust and understand the rationale behind the predictions. Additionally, extending the framework to support federated learning architectures could enhance data privacy and security while enabling collaborative learning across multiple healthcare institutions. Such enhancements would position the framework as a cutting-edge solution for both real-time applications and long-term strategic healthcare planning. By addressing the critical needs of modern healthcare systems—accuracy, speed, scalability, and adaptability—the proposed HDBN and CAEN framework demonstrates its potential to transform real-time disease prediction and improve overall patient outcomes.

### Computational complexity and optimization strategies

The computational complexity of the proposed framework was analyzed to assess its practicality for large-scale deployments. Key metrics, including training time, inference time, and GPU memory usage, were recorded during the experiments. On average, the training phase for each dataset took approximately 4.5 h on a high-performance server with an NVIDIA GPU (24 GB memory), while inference required 200 ms per sample. The peak GPU memory usage during training was approximately 18 GB, highlighting the resource-intensive nature of the framework, particularly during the training phase. To mitigate computational overhead and enhance scalability, several optimization strategies can be explored. Model pruning techniques can be employed to reduce the number of parameters while maintaining performance. Quantization approaches, which involve representing model weights and activations with lower precision (e.g., INT8), can significantly reduce memory usage and inference latency. Furthermore, techniques like knowledge distillation could enable the deployment of lightweight student models trained to replicate the performance of the original framework. Future implementations may also leverage distributed training and inference pipelines, enabling the model to process larger datasets across multiple devices efficiently. By incorporating these strategies, the framework’s computational demands can be optimized, making it more suitable for large-scale and real-time applications in healthcare and other domains.

### Ethical considerations and clinical challenges

The proposed framework acknowledges the critical importance of aligning with clinical workflows and addressing regulatory requirements to ensure safe and effective adoption in healthcare settings. Two key aspects—model interpretability for healthcare providers and patient data privacy—are crucial for its successful deployment and integration. One of the primary challenges in applying deep learning models in clinical environments is ensuring interpretability for healthcare providers. Black-box models can hinder trust and acceptance, as clinicians need to understand the rationale behind predictions to make informed decisions. To address this, future work will incorporate explainable AI (XAI) techniques, such as feature importance mapping and visual explanations, to make the model's decision-making process transparent. For instance, saliency maps could help clinicians visualize critical areas in medical images influencing the predictions, while feature attribution methods could identify key variables in tabular datasets.

Ensuring compliance with regulatory frameworks such as the Health Insurance Portability and Accountability Act (HIPAA) and the General Data Protection Regulation (GDPR) is paramount in handling sensitive patient data. The framework's current implementation emphasizes secure data handling through encryption and anonymization techniques [38]. However, additional measures, such as federated learning, could be explored to enable model training across multiple institutions without sharing raw data, preserving privacy while enhancing model generalizability. For seamless integration into clinical settings, the framework must align with established workflows. The modular design of the proposed system allows for flexible integration into existing electronic health record (EHR) systems and clinical decision-support tools. By enabling real-time predictions and offering interpretable insights, the framework could support time-sensitive applications such as early diagnosis, treatment planning, and monitoring of critical conditions. In conclusion, addressing these ethical considerations and clinical challenges is essential for translating the proposed framework from research to practical applications. Future enhancements will focus on refining interpretability mechanisms, ensuring compliance with privacy regulations, and fostering adaptability to diverse clinical environments.

### Limitations and future directions

Our proposed framework provides great progress in multi-disease prediction while certain limitations point out promising ways of improvement. Firstly, the computational complexity of the Hybrid Deep Belief Network (HDBN) and Custom Adaptive Ensemble Network (CAEN) increases with the inclusion of large-scale and high-dimensional datasets. This helps in robust feature extraction and prediction accuracy, but would not be desirable in environments with limited resources for computing. The future work is to optimize the computational efficiency by making use of lightweight model architectures and distributed computing. Secondly, this framework employs a number of intensive preprocesses including hierarchical contrast normalization and multi-scale region enhancement to improve data quality but this comes at the cost of increased processing time in real-time situations. In subsequent iterations of this process, these steps will be streamlined or the removal of some computation with an automated preprocessing pipeline with minimal computational overhead will be explored. Moreover, while the evolutionary optimization techniques effectively enhance model performance, their inherent stochastic nature may occasionally result in suboptimal parameter selection. Future research will address this by integrating hybrid optimization methods that combine deterministic and heuristic approaches for consistent results. Finally, the framework’s performance has been evaluated across a diverse range of datasets, but its adaptability to entirely new disease domains or modalities remains to be fully explored. Future work will include incorporating transfer learning and continual learning techniques to enable the model to adapt dynamically to evolving medical datasets. These limitations, while reflective of the current state of the framework, pave the way for promising avenues of research, ensuring the continuous evolution of the proposed system toward greater scalability, efficiency, and real-world applicability.

While the proposed framework demonstrates robust performance across four Kaggle datasets—covering diabetes prediction, breast cancer diagnosis, Parkinson’s disease detection, and heart disease prediction—it is essential to acknowledge potential limitations and biases associated with these datasets. Kaggle datasets, though widely used and publicly accessible, may not fully represent the complexities and variabilities encountered in real-world clinical environments. These datasets often undergo preprocessing, normalization, and curation, which may remove noise and irregularities present in electronic health records (EHR) or multi-center studies. As a result, the framework’s performance might be influenced by the standardized and homogeneous nature of these datasets, potentially leading to optimistic evaluations. In practical scenarios, data from EHR systems or multi-center studies are characterized by diverse patient demographics, varying data quality, and heterogeneous sources, including imaging modalities, lab results, and unstructured clinical notes. Such variability could introduce challenges, such as handling missing values, inconsistent feature representations, and biases arising from site-specific practices. To address these limitations, future work will focus on validating the framework on more diverse and heterogeneous datasets, including real-world EHR data and multi-center studies. Efforts will also include implementing advanced techniques such as transfer learning and domain adaptation to generalize the model across varied clinical settings. By incorporating these strategies, the proposed framework can ensure adaptability, robustness, and broader applicability in real-world healthcare scenarios.

## Conclusion & future work

In this study, we introduced a robust hybrid framework combining a Hybrid Deep Belief Network (HDBN) and a Custom Adaptive Ensemble Network (CAEN) to tackle critical challenges in classification and segmentation tasks across diverse datasets. The framework’s innovative design integrates advanced feature extraction, dynamic prediction aggregation, and robust optimization mechanisms, ensuring adaptability, scalability, and exceptional performance. Extensive evaluations demonstrated that the proposed framework consistently outperformed existing methods, achieving peak accuracy of 93 %, precision of 87 %, specificity of 95 %, and recall of 91 %. Advanced metrics, including a False Positive Rate of 0.0500, False Discovery Rate of 0.0515, and a Matthews Correlation Coefficient of 0.8932, validated the system's reliability, while confusion matrix analysis highlighted its ability to effectively control false positives and false negatives across diverse scenarios. The contributions of this work include the design of a novel hybrid architecture, superior performance enhancements, and a comprehensive evaluation of its robustness and reliability. Looking forward, this research paves the way for several future advancements, including integrating explainable AI techniques to enhance transparency and interpretability, incorporating continual learning strategies to improve adaptability in dynamic environments, and extending the framework to multi-modal datasets and real-time applications for further validation of its scalability and applicability to complex real-world scenarios. By addressing pressing challenges in healthcare data classification and segmentation, this study establishes a benchmark for AI-driven decision-making in healthcare and beyond.

## Ethics statements

In this Manuscript no, human participants or animals their data or biological material, are not involved.

## CRediT authorship contribution statement

**G. Prabaharan:** Methodology, Writing – original draft. **S.M. Udhaya Sankar:** Visualization, Investigation, Software. **V. Anusuya:** Conceptualization, Methodology, Writing – original draft. **K. Jaya Deepthi:** Data curation, Software, Validation, Investigation. **Rayappan Lotus:** Methodology, Writing – review & editing. **R. Sugumar:** Writing – review & editing, Investigation.

## Declaration of competing interest

The authors declare that they have no known competing financial interests or personal relationships that could have appeared to influence the work reported in this paper.

## Data Availability

No data was used for the research described in the article.
